# Myxoma of the Nasal Fossa of a Horse

**Published:** 1903-01

**Authors:** John J. Repp

**Affiliations:** Ames, Iowa


### REPORTS OF CASES.

### MYXOMA OF THE NASAL FOSSA OF A HORSE.

By John J. Repp, V.S.,
AMES, IOWA.

On September 9, 1899, there was brought to my clinic at the Veterinary Hospital, Iowa State College, a brown gelding, six years old, in good flesh, suffering considerable dyspnœa owing to the complete occlusion of the left nasal fossa by a tumor. The tumor protruded for an inch or more below the nostril and extended up the fossa farther than the finger could reach. The tumor was soft and jelly-like, although enclosed in a strong, translucent capsule. It could be pushed aside and its shape changed readily by manipulation. The horse was cast upon the grass, chloroform anæsthesia induced, and a slit was made through the skin covering the false nostril extending upward to the angle formed by the nasal and the superior maxillary bone, and downward nearly to the free border of the nostril. This incision was made in order to gain more ready access to the point of attachment of the tumor. Partly by traction and partly by cutting with a long-handled bistoury, blindly, as best one might, the tumor was brought away. As nearly as could be ascertained, the tumor had its attachment about the middle of the nasal septum by a comparatively small base. The mass weighed nine ounces.

On microscopic examinations of sections from it the structure was seen to be that of the myxoma, to wit: the large stellate connective tissue cells widely separated by an almost amorphous intercellular substance.

The incision in the wall of the fossa was closed by interrupted silk sutures and a collodion dressing applied. It healed promptly without suppuration. The after-treatment of the fossa consisted in daily douches with 1 per cent. lysol solution. There was very little discharge and recovery was rapid and uneventful. The animal was discharged September 16th. A letter from the owner, dated January 22, 1901, in response to an inquiry from me contains the following statement: "The horse has been doing fine since you removed the tumor." I infer, therefore, that there was no recurrence.

---

Dr. W. H. Dalrymple, of Baton Rouge, La., fills the rôle of Secretary-Treasurer for the Louisiana Stockbreeders' Association.

